# Preclinical Evaluation of a Chitosan-Based Field Hemostatic Sponge in Arterial Bleeding Models with and Without Aspirin Administration

**DOI:** 10.3390/jfb17070349

**Published:** 2026-07-18

**Authors:** Quang Thai Vu, Hoang Ngan Nguyen, Thi Xoan Le, Thi Thuy Trang Do, Trong Hung Mai

**Affiliations:** 1 National Institute of Medicinal Materials, Hanoi 100000, Vietnam; xoanle@nimm.org.vn; 2 Hanoi Obstetrics and Gynecology Hospital, Hanoi 100000, Vietnam; bacsymaitronghung@gmail.com; 3 Department of Pharmacology, Institute of Pharmacy Education, Vietnam Military Medical University, Hanoi 100000, Vietnam; nganvmu@gmail.com; 4 Joint Vietnam-Russia Tropical Science and Technology Research Center, Hanoi 100000, Vietnam; thuytrang.ttndvn@gmail.com

**Keywords:** Field Hemostatic Sponge (FHS), chitosan, hemostatic biomaterial, in vivo bleeding model, rabbit ear incision, femoral artery puncture, aspirin

## Abstract

Background/Objectives: This study evaluated the hemostatic efficacy of a chitosan-based field hemostatic sponge (FHS), the first hemostatic product of this type manufactured in Vietnam, across bleeding models of varying injury severity under both normal physiological conditions and after aspirin administration. Methods: An in vivo experimental study was conducted using two models: (1) a rabbit ear incision model in New Zealand White rabbits and (2) a femoral artery puncture model in Wistar rats and New Zealand White rabbits. Animals were randomly allocated to six groups: medical gauze (control), FHS, Axiostat^®^ (reference material), and three corresponding aspirin-treated groups (*n* = 10 per group for non-aspirin conditions; *n* = 6 per group for aspirin-treated rabbits; *n* = 10 per group for aspirin-treated rats). Primary outcomes were time to hemostasis (seconds) and blood loss (mg). Results: In the rabbit ear incision model, FHS reduced time to hemostasis by 57.6% and blood loss by 69.9% compared with medical gauze (*p* < 0.001). In the femoral artery puncture model, reductions were 62–65% and 75–76% (*p* < 0.001). Under aspirin treatment, FHS maintained significant hemostatic advantages over gauze (55–66% reduction in time to hemostasis and 69–76% reduction in blood loss; *p* < 0.01). No significant difference was observed between FHS and Axiostat^®^ in any condition (*p* > 0.05). Conclusions: FHS demonstrated potent and consistent hemostatic efficacy across multiple injury severities and animal species; the hemostatic efficacy of FHS was maintained after aspirin administration, indicating that the material’s efficacy is not entirely dependent on platelet function. These findings support its potential clinical application in hemorrhage control, including in patients with coagulation disorders or receiving antiplatelet therapy.

## 1. Introduction

Acute hemorrhage resulting from trauma, accidents, or vascular injury is one of the leading causes of preventable death worldwide. In many cases, fatalities occur not from the severity of the initial injury but from uncontrolled blood loss that is not managed promptly. High-pressure arterial bleeding, in particular, progresses rapidly, is difficult to control, and requires immediate and effective on-site intervention. The development of hemostatic materials capable of rapid, safe, and easy-to-use bleeding control has therefore become an important research priority in biomedical engineering and surgical medicine [1].

Several topical hemostatic agents are currently used clinically, including products derived from gelatin, collagen, oxidized cellulose, and inorganic minerals. These materials have demonstrated efficacy in a range of surgical applications, including parenchymal organ surgery, cardiovascular surgery, and control of diffuse bleeding. However, their hemostatic performance can vary depending on the type of injury and clinical context; controlling high-pressure arterial bleeding, deep wounds of complex geometry, or trauma under prehospital conditions remains challenging and requires materials capable of rapid hemostasis, ease of use, and broad applicability. Natural polysaccharide-based biomaterials have attracted considerable interest in this context. Among these, chitosan is one of the most extensively studied, owing to its rapid hemostatic activity, good biocompatibility, biodegradability, inherent antibacterial properties, and potential for formulation into diverse hemostatic products [1].

Chitosan is a polysaccharide derived from the deacetylation of chitin. Its positively charged amino groups allow electrostatic interaction with the negatively charged membranes of erythrocytes and platelets, promoting cellular aggregation and thrombus formation [2,3]. Additionally, chitosan can rapidly absorb plasma, concentrate cellular blood components at the injury site, and form a physical barrier that mechanically supports hemostasis. Recent studies have demonstrated that chitosan sponges with three-dimensional microporous architectures exhibit rapid blood absorption, providing a favorable microenvironment for coagulation and superior hemostatic performance compared with many conventional materials [4]. Reviews by Zhang et al. [5] and Liu et al. [4] have comprehensively summarized the mechanisms and clinical potential of chitosan-based hemostatic sponges. The functional performance of such sponges is critically determined by their physicochemical parameters—including degree of deacetylation (DDA), microporous pore size, and fluid absorption capacity—which collectively govern blood uptake rate, coagulation factor concentration efficiency, and the structural integrity of the hemostatic construct.

Recent studies have further confirmed this potential. Zhou et al. [6] reported that carboxymethyl chitosan sponge demonstrated rapid blood absorption and improved hemostatic performance in a penetrating trauma model. Yan et al. [7] found that a keratin/chitosan composite sponge significantly reduced time to hemostasis and blood loss in an uncontrolled hemorrhage model. Wang et al. [8] demonstrated that a chitosan-based antibacterial sponge outperformed commercial gelatin sponge across multiple animal injury models.

Aspirin is among the most widely prescribed antiplatelet agents for the prevention and treatment of cardiovascular disease. It irreversibly inhibits cyclooxygenase-1 (COX-1) in platelets, reducing thromboxane A2 (TXA2) synthesis and impairing platelet aggregation, thereby prolonging bleeding time [9,10]. Patients receiving aspirin are at increased risk of prolonged and difficult-to-control bleeding following trauma or surgery. However, evidence evaluating chitosan-based hemostatic materials under aspirin administration in animal models remains limited, particularly in arterial injury models, a clinically important research gap.

Furthermore, advanced hemostatic products are predominantly imported at high cost, whereas domestic chitin/chitosan resources derived from seafood-processing by-products are abundantly available in Vietnam, providing favorable conditions for the development of domestic biomedical products.

Based on the foregoing, this study evaluated the hemostatic efficacy of FHS, a chitosan-based field hemostatic sponge produced in Vietnam. Field Hemostatic Sponge (FHS) refers to a hemostatic sponge designed primarily for controlling bleeding at the point of injury and during prehospital emergency care, prior to the patient’s transfer to a medical facility for definitive treatment. The present study evaluated FHS across arterial bleeding models of varying injury severity, under both normal physiological conditions and after aspirin administration. This is the first study in Vietnam to simultaneously evaluate a domestically produced chitosan-based hemostatic sponge across three preclinical scenarios: the rabbit ear incision model and the femoral artery puncture model in both Wistar rats and New Zealand White rabbits, benchmarked against medical gauze and the internationally established reference Axiostat^®^. The study addressed three specific objectives: (1) to characterize FHS hemostatic performance across injury models differing in vascular severity and species hemodynamic profiles; (2) to determine whether hemostatic efficacy is maintained after aspirin administration, and to assess the extent to which the hemostatic mechanism is not entirely dependent on TXA2-mediated platelet aggregation; and (3) to generate inter-species comparative data (rats vs. rabbits) relevant to preclinical model selection for hemostatic biomaterial evaluation. The findings are intended to provide multidimensional experimental evidence supporting the clinical translation of FHS as a functional hemostatic biomaterial, particularly for difficult-to-control arterial hemorrhage and for use in patients receiving antiplatelet therapy, while contributing to the rational design of hemostatic agents whose activity is not entirely dependent on platelets.

## 2. Materials and Methods

### 2.1. Study Subjects, Materials, and Equipment

#### 2.1.1. Test Materials

The test material was FHS, a chitosan-based sponge composed of deacetylated chitosan with a degree of deacetylation (DDA) of 73.6–87.7%, pore size ranging from 40 to 170 µm, fluid absorption capacity of 24.8 g/g, and tensile strength of 23.22 MPa. The sponge was manufactured by the Vietnam–Russia Tropical Center, Ministry of National Defence, Vietnam, in compliance with the institutional technical specification (Supplementary Table S1). Each sponge sheet measured 8 cm × 8 cm and weighed 2.8–3.4 g, packaged in sealed sterile aluminum pouches. Sheets were cut to 2 cm × 2 cm or 3 cm × 5 cm as required. The physicochemical characteristics of FHS and Axiostat^®^ were characterized and compared in the present study (see Section 2.1.4); key parameters are presented in Table 1.

**Figure 1:**
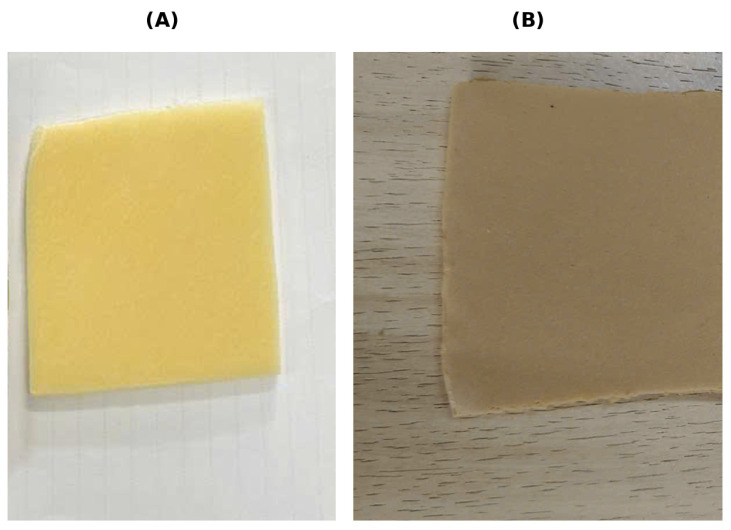
Representative images of Axiostat^®^ (**A**) and FHS (**B**).

Axiostat^®^ (Axio Biosolutions Pvt. Ltd., Ahmedabad, India), a commercially available chitosan-based hemostatic dressing used internationally for bleeding control, served as the reference material. Each sheet measured 8 cm × 8 cm and was cut to 2 cm × 2 cm or 3 cm × 5 cm as required. Comparative characterization of both materials is presented in Table 1. Representative photographs of Axiostat® and FHS are shown in Figure 1.

#### 2.1.2. Experimental Animals

The study used (1) healthy Wistar rats weighing 180–220 g of both sexes (non-pregnant females) and (2) healthy New Zealand White rabbits weighing 2.5–3.5 kg of both sexes (non-pregnant females). All animals were supplied by the Department of Experimental Animal Supply, Military Medical University, Vietnam. Animals were housed under standard laboratory conditions for at least one week before experimentation and were provided with standard chow and ad libitum access to water.

#### 2.1.3. Equipment and Chemicals

Standard surgical instruments (scalpel, scissors, hemostats), sterile gauze pads, adhesive tape, animal hair clippers, and a digital stopwatch were used. The antiplatelet agent administered was aspirin 100 mg tablets (Traphaco, Hanoi, Vietnam) [11,12]. Additional reagents included pentobarbital sodium (anesthetic; 40 mg/kg intraperitoneally) [13,14], 0.9% NaCl solution, and other consumables for the experimental procedures.

#### 2.1.4. Physicochemical Characterization

**Figure 2:**
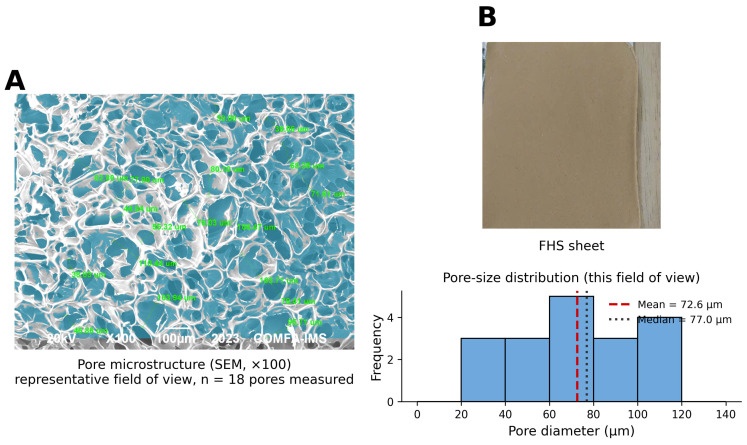
SEM pore microstructure (**A**) and macroscopic appearance with pore-size distribution (**B**) of FHS. Panel A shows a representative field of view (*n* = 18 pores measured), illustrating pore morphology; it is intended to depict the microstructure of a single representative region rather than a whole-sample size distribution, which is reported over multiple fields as the range given in Table 1.

The degree of deacetylation (DDA) of FHS and Axiostat^®^ was determined using three independent methods: ^1^H nuclear magnetic resonance (^1^H-NMR) spectroscopy, acid–base titration, and UV spectrophotometry. Tensile strength was measured using a calibrated tensiometer on standardized sponge strips. Fluid absorption capacity was determined gravimetrically as the mass of 0.9% NaCl solution absorbed per gram of dry sponge. Pore size was measured from scanning electron microscopy (SEM) images acquired using a JSM-6510LV scanning electron microscope (JEOL Ltd., Tokyo, Japan) at ×100 magnification using ImageJ v1.54 (National Institutes of Health, Bethesda, MD, USA) software, based on multiple fields of view across sponge samples. All measurements were performed by the authors in the present study; comparative results for FHS and Axiostat^®^ are presented in Table 1, and the institutional technical specification for FHS (11 quality-control parameters) is provided in Supplementary Table S1. A representative SEM image of the FHS pore microstructure, together with a macroscopic photograph of the sponge and the corresponding pore-size distribution, is shown in Figure 2.

### 2.2. Methods

#### 2.2.1. Study Design

This study employed an in vivo experimental design to evaluate hemostatic efficacy and investigate the potential mechanism of action of FHS. Two bleeding injury models were used: (1) a rabbit ear incision model (New Zealand White rabbits only) and (2) a femoral artery puncture model (both Wistar rats and New Zealand White rabbits). Hemostatic efficacy was assessed based on time to hemostasis and blood loss. A standardized experimental design was applied across all models to ensure consistency and comparability.

#### 2.2.2. Rationale and Principles of the Experimental Models

Following vascular injury, platelets are activated and aggregate via thromboxane A2 (TXA2), forming the primary hemostatic plug. Aspirin irreversibly inhibits COX-1 in platelets, reducing TXA2 synthesis, impairing platelet activation and aggregation, and prolonging bleeding time [9,10,12,13]. Aspirin was therefore administered to establish an in vivo model of increased bleeding risk for evaluating the hemostatic efficacy of FHS under pharmacologically challenged hemostatic conditions.

#### 2.2.3. Experimental Procedures

##### Animal Preparation

Animals were randomly allocated to two groups: a non-aspirin (control) group and an aspirin-treated group. Animals in the aspirin group received aspirin orally for five consecutive days (rats: 9 mg/kg/day; rabbits: 4 mg/kg/day), based on human therapeutic doses for cardiovascular prevention [15] converted to animal equivalent doses using established interspecies allometric scaling [16]. Experiments were performed 2 h after the final dose. Animals were anesthetized by intraperitoneal injection of sodium pentobarbital (40 mg/kg) [13,14].

Sample sizes were determined in accordance with the 3R principles of laboratory animal research. Non-aspirin groups comprised *n* = 10 animals per group, consistent with comparable hemostasis studies [7,17,18], to evaluate the primary hemostatic efficacy of FHS under physiological conditions. Aspirin-treated groups in rabbits comprised *n* = 6 animals per group, as this was a supplementary assessment under aspirin administration rather than the primary outcome; reducing the number of the larger species remained scientifically justified in accordance with the Reduction principle and is consistent with comparable hemostasis studies [7,18,19,20,21]. Aspirin-treated groups in rats comprised *n* = 10, equal to the non-aspirin group.

##### Experimental Models

(1) Rabbit ear incision model

Rabbits were randomly allocated to six groups (*n* = 10 per group for non-aspirin; *n* = 6 per group for aspirin-treated): medical gauze, FHS, Axiostat^®^, aspirin + medical gauze, aspirin + FHS, and aspirin + Axiostat^®^. After anesthesia, the ear was depilated and moistened with 0.9% NaCl. A standardized full-thickness incision was made using a No. 11 Bard-Parker scalpel blade, perpendicular to and transecting the marginal ear artery and vein. The first drop of blood was blotted away; the hemostatic material (FHS, Axiostat^®^, or medical gauze; 2 cm × 2 cm) was immediately applied to the incision site, and gentle manual compression was maintained continuously, by the same trained operator, until complete hemostasis was achieved. The procedure was adapted from Cheng et al. [17].

(2) Femoral artery puncture model

Animals were randomly allocated to six groups using the same structure as above. After anesthesia, the right inguinal region was depilated and sterilized, and a skin incision of approximately 5 cm was made to expose a 3–4 cm segment of the femoral artery. Arterial injury was created using a 23G needle by puncturing through one vessel wall at a defined anatomical location, establishing a standardized arterial hemorrhage model. The hemostatic material (3 cm × 5 cm) was immediately applied to the injury site. Gentle, continuous compression was maintained by the same trained operator throughout the observation period, using direct hand pressure or a clamp to hold the sponge against the injury site. The procedure was based on the model described by Kim et al. [18] with modifications. Representative intraoperative photographs of the bleeding models are provided in Supplementary Figure S1.

##### Outcome Assessment

Time to hemostasis was defined as the interval from application of the hemostatic material to complete cessation of bleeding, confirmed by the absence of visible bleeding or oozing for at least 10 consecutive seconds while the material remained in place under continuous gentle compression. Animals in which bleeding persisted beyond 10 min were classified as hemostatic failures; the actual elapsed time was nonetheless recorded for all animals throughout the observation period. All animals were monitored until the end of the procedure to record time to hemostasis, blood loss, and survival status. Following initial hemostasis, the wound site was observed for a further 30 min to assess for rebleeding, and animals were monitored for 24 h post-procedure in accordance with institutional animal-ethics requirements.

For the gauze group, blood loss was determined gravimetrically as the weight difference in pre-weighed gauze pads before and after blood absorption. For the chitosan sponge groups (FHS and Axiostat^®^), the sponge was applied directly to the bleeding site while a separate pre-weighed gauze pad was used concurrently to absorb any blood leaking around the sponge margin. After hemostasis was achieved, both the sponge and the auxiliary pad were reweighed, and total blood loss was calculated as the sum of the weight gained by the sponge and the auxiliary pad.

Animals were coded prior to intervention. Only the operator administering aspirin was aware of treatment allocation; the surgeon and the evaluators recording time to hemostasis and weighing blood loss were blinded to group assignment (single-blind design), minimizing observer bias.

Generative artificial intelligence was not used in this research.

### 2.3. Statistical Analysis

Data were analyzed using Microsoft Excel 2010 and IBM SPSS Statistics v20.0 (IBM Corp., Armonk, NY, USA). Results are presented as mean ± SD. Between-group comparisons were performed using Welch’s independent-samples *t*-test. A two-tailed *p* < 0.05 was considered statistically significant. Experiments were conducted with 6–10 independent replicates depending on the animal model.

## 3. Results

### 3.1. Rabbit Ear Incision Model

Table 2 summarizes time to hemostasis and blood loss for medical gauze, FHS, and Axiostat^®^ in the rabbit ear incision model, under normal and aspirin-treated conditions.

As shown in Table 2, under physiological conditions, both FHS and Axiostat^®^ significantly reduced time to hemostasis (68.8 s and 69.1 s vs. 162.1 s for medical gauze; *p* < 0.001) and blood loss (62.6 mg and 63.7 mg vs. 208.3 mg; *p* < 0.001), corresponding to reductions of 57.4–69.9%. The relatively low SD of these outcomes (approximately 5–10% of the mean) reflects the high degree of standardization applied throughout the protocol, including a single trained operator per model, a standardized injury procedure, and blinded outcome assessment.

Under aspirin treatment, time to hemostasis and blood loss increased in all groups (1.81–2.48-fold compared with non-aspirin conditions). Nevertheless, FHS and Axiostat^®^ maintained significantly superior hemostatic performance compared with medical gauze (131.3 s and 132.5 s vs. 293.7 s; 155.2 mg and 153.8 mg vs. 504.8 mg; *p* < 0.01). The relative reductions remained stable (54.9–57.6% in time to hemostasis; 69.3–69.9% in blood loss), suggesting that the hemostatic mechanism of FHS is not wholly dependent on platelet aggregation function.

No statistically significant difference was observed between FHS and Axiostat^®^ in either condition (*p* > 0.05).

### 3.2. Femoral Artery Puncture Model

Table 3 summarizes time to hemostasis and blood loss for medical gauze, FHS, and Axiostat^®^ in the femoral artery puncture model in Wistar rats, under normal and aspirin-treated conditions.

As shown in Table 3, FHS and Axiostat^®^ significantly reduced time to hemostasis (171.5 s and 175.1 s vs. 487.8 s; 64.1–64.8% reduction; *p* < 0.001) and blood loss (207.9 mg and 211.2 mg vs. 864.6 mg; 75.6–76.0% reduction; *p* < 0.001) under physiological conditions. Under aspirin treatment, all groups showed 1.48–1.55-fold increases in time to hemostasis and 5.92–6.15-fold increases in blood loss; however, the superiority of FHS and Axiostat^®^ over medical gauze was maintained (*p* < 0.01). Relative reductions remained stable at 64–66% (time) and 75% (blood loss).

Table 4 summarizes time to hemostasis and blood loss for medical gauze, FHS, and Axiostat^®^ in the femoral artery puncture model in New Zealand White rabbits, under normal and aspirin-treated conditions.

As shown in Table 4, FHS and Axiostat^®^ significantly reduced time to hemostasis (192.8 s and 187.2 s vs. 508.8 s; 62–63%; *p* < 0.001) and blood loss (~75%; *p* < 0.001). Under aspirin treatment, time to hemostasis increased ~1.7-fold and blood loss ~5-fold in all groups; relative reductions remained stable (61.8–62.5% in time to hemostasis; 75.2–75.6% in blood loss; *p* < 0.001 vs. medical gauze). No significant difference was observed between FHS and Axiostat^®^ (*p* > 0.05).

**Figure 3:**
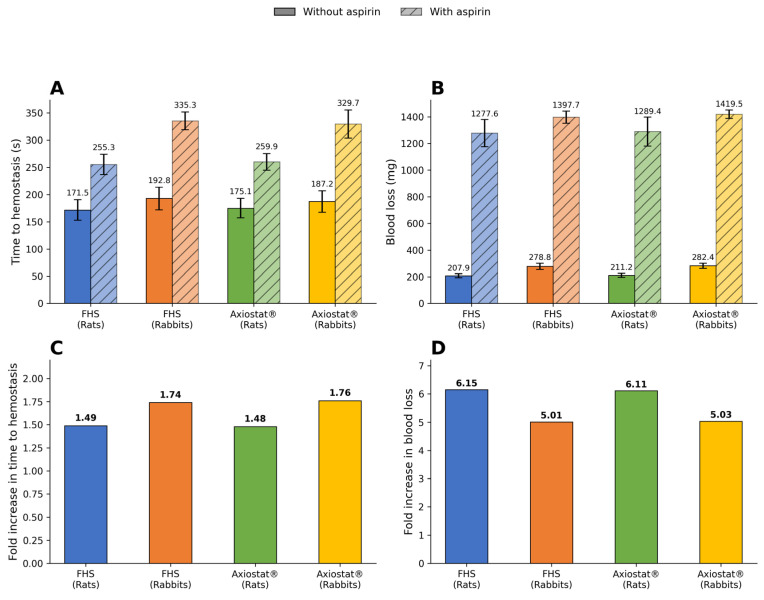
Time to hemostasis, blood loss, and fold increase after aspirin pretreatment in the femoral artery puncture model (rats vs. rabbits; FHS vs. Axiostat^®^). (**A**) Time to hemostasis; (**B**) blood loss; (**C**) fold increase in time to hemostasis; (**D**) fold increase in blood loss.

As shown in Figure 3, rabbits exhibited higher absolute values for both time to hemostasis and blood loss than rats under both conditions, reflecting greater femoral artery diameter and higher blood flow pressure. Under aspirin treatment, time to hemostasis in the FHS and Axiostat^®^ groups increased 1.74–1.76-fold in rabbits vs. 1.48–1.49-fold in rats, indicating greater bleeding sensitivity to aspirin administration in rabbits. The fold increase in blood loss was higher in rats (6.11–6.15) than in rabbits (5.01–5.03), reflecting differences in baseline blood loss between the two species.

## 4. Discussion

The study design employed two models of progressively increasing vascular injury severity: the rabbit ear incision model simulates superficial (arteriolar and capillary) injury with low invasiveness and high reproducibility; the femoral artery puncture model simulates deep high-pressure arterial hemorrhage, representative of severe vascular injury that is difficult to manage clinically. The concurrent use of two models of different injury severity, in two animal species with distinct hemodynamic characteristics, enhances the robustness and translational relevance of the findings.

### 4.1. Rabbit Ear Incision Model

Under physiological conditions, FHS achieved rapid and effective hemostasis, reducing time to hemostasis by 57.6% and blood loss by 69.9% compared with medical gauze (Table 2). This performance is primarily attributed to the sponge’s capacity for rapid absorption of blood fluid, mechanical scaffold provision, concentration of coagulation factors, and acceleration of fibrin network formation. These findings are consistent with Cheng et al. [17], who reported that a norfloxacin-grafted chitosan sponge reduced time to hemostasis from 127.55 s to 49.75 s and blood loss from 0.233 g to 0.07 g vs. gauze in a rabbit ear arterial bleeding model. Pan et al. [22] similarly demonstrated that a PVA-HLC-T80 chitosan hydrogel dressing achieved the shortest time to hemostasis (55.25 s) and lowest blood loss (21.30 mg) among tested materials. Wang et al. [23] also demonstrated that an insect chitosan-based self-gelling powder produced rapid hemostasis with significantly reduced blood loss in a rabbit ear artery model.

Under aspirin treatment, time to hemostasis and blood loss increased in all groups (1.81–2.48-fold), consistent with aspirin’s irreversible COX-1 inhibition and consequent reduction in TXA2 synthesis, leading to impaired platelet activation, delayed primary hemostatic plug formation, and reduced clot stability [9,10]. Kelton and Carter [24] similarly reported that aspirin produced smaller, less stable hemostatic plugs in a rabbit ear puncture model, prolonging bleeding time. Despite these effects, FHS maintained significant hemostatic superiority (55.3% reduction in time to hemostasis and 69.3% reduction in blood loss vs. gauze), with stable relative reductions between aspirin-treated and non-aspirin conditions, suggesting that the hemostatic mechanism is not wholly dependent on platelet activation.

Darya et al. [25] confirmed that the rabbit ear hemorrhage model provides high reproducibility and controllability, making it suitable for evaluating topical hemostatic agents in superficial vascular injury and for assessing efficacy under aspirin administration. These findings collectively suggest that FHS has clinical potential as a hemostatic biomaterial in scenarios involving impaired platelet function, including patients receiving antiplatelet therapy.

### 4.2. Femoral Artery Puncture Model

In the femoral artery puncture model, FHS effectively controlled high-pressure arterial hemorrhage, achieving 62–65% reductions in time to hemostasis and 75–76% reductions in blood loss relative to medical gauze (Table 3 and Table 4). The hemostatic mechanism involves the three-dimensional microporous architecture of the sponge, which enables the rapid absorption of blood fluid and the concentration of erythrocytes, platelets, and coagulation factors at the injury site, promoting stable clot formation [26,27]. The cationic surface of chitosan further interacts electrostatically with negatively charged erythrocyte and platelet membranes, driving cellular aggregation and facilitating primary hemostatic plug formation [7,28,29].

These results are consistent with published reports on chitosan-based hemostatic materials in arterial models. Fan et al. [30] reported that chitosan hydrogel reduced time to hemostasis from 7.3 to 5.4 min in a rat femoral artery model. Wang et al. [31] demonstrated that chitosan fibers and sponges reduced bleeding time to 4.69–5.40 min vs. 7.33 min for gauze. Huang et al. [14] reported that a protonated chitosan sponge (PCS) significantly reduced blood loss in a rat femoral artery coagulopathy model. Elsabahy and Hamad [32] demonstrated that a chitosan/kaolin nanocomposite reduced time to hemostasis from 3306 s to 294 s and blood loss from 31,100 mg to 3500 mg in rabbit vascular injury models.

Under aspirin treatment, time to hemostasis and blood loss increased substantially in both species (1.49–1.74-fold and 5.01–6.15-fold, respectively), consistent with aspirin-mediated irreversible inhibition of platelet COX-1 and consequent TXA2 deficiency [9,10]. The disproportionately greater increase in blood loss relative to time to hemostasis indicates that aspirin not only delays primary hemostatic plug formation but also compromises clot mechanical stability, consistent with the findings of Kelton and Carter [24].

Despite aspirin administration, FHS reduced time to hemostasis by 61.8–66.3% and blood loss by 75.0–75.6% vs. gauze across both species and conditions. This maintenance of hemostatic efficacy after aspirin administration suggests a procoagulant mechanism that does not rely solely on TXA2-dependent platelet aggregation. Upon contact with blood, the porous architecture and cationic nature of chitosan promote rapid fluid absorption, erythrocyte and platelet concentration, coagulation factor retention, and mechanical occlusion at the injury site [14,17,27,29]. Additionally, chitosan has been shown to activate Toll-like receptor 2 (TLR2) on platelets, a signaling pathway not inhibited by aspirin or clopidogrel, thereby sustaining platelet adhesion and activation even under COX-1 inhibition [14,33].

Compared with other hemostatic materials, the mechanism of action varies according to composition and structure. Gelatin-based materials can promote hemostasis through rapid blood absorption, provision of a scaffold for clot formation, and support of platelet adhesion and activation [34]. Tranexamic acid-grafted nanocellulose materials combine absorptive concentration of blood components with antifibrinolytic activity to promote clot formation and stabilization [35], whereas catechol-modified cotton gauze enhances hemostasis primarily through tissue adhesion and control of blood spread at the injury site [36]. Dextran-derivative sponges achieve rapid hemostasis primarily through strong wet-tissue adhesion rather than direct platelet activation [37]. In contrast, chitosan carries a positive charge that enables electrostatic interaction with erythrocytes and platelets, promoting clot formation and maintaining hemostatic efficacy even after aspirin administration [14,33]. These properties make chitosan-based sponges promising candidates as active hemostatic agents for topical use [38,39]. Notably, FHS demonstrated hemostatic performance statistically equivalent to Axiostat^®^ across all three models and both conditions—in rats and rabbits, under normal physiological conditions and after aspirin administration—supporting its clinical potential as a functional hemostatic biomaterial with performance comparable to a commercially established reference product.

Regarding inter-species differences, rabbits exhibited higher absolute values for time to hemostasis and blood loss than rats under both conditions (Figure 3), reflecting greater femoral artery diameter, higher cardiac output, and higher intravascular pressure—hemodynamic factors that increase the mechanical challenge on clot stability [38,39]. Known differences in platelet membrane glycoprotein composition and coagulation parameters between species [40,41] and the modulatory role of erythrocytes in hemostatic clot mechanics [42] may also contribute. Under aspirin treatment, the fold increase in time to hemostasis was greater in rabbits (1.74–1.76-fold vs. 1.48–1.49-fold in rats; Figure 3), consistent with greater rabbit platelet sensitivity to COX-1 inhibition; the higher arterial flow velocity in rabbits further intensifies clot instability after aspirin administration [15,16]. The fold increase in blood loss was higher in rats (6.11–6.15-fold vs. 5.01–5.03-fold in rabbits), attributable to the lower baseline blood loss in rats; aspirin administration has been shown to substantially destabilize thrombus formation under arterial flow conditions in rodent models [43].

### 4.3. Limitations

Several limitations of this study should be acknowledged. First, physicochemical characterization of FHS was performed during product development; the comparative parameters in Table 1 were measured in the present study, FHS met all 11 acceptance criteria of the institutional technical specification (Supplementary Table S1), and comparative in vitro hemostatic activity against Axiostat^®^ has been reported separately [44]. In-house biocompatibility testing further indicated FHS to be non-cytotoxic to L929 cells (85.32–89.47% viability), non-hemolytic (1.83%), and free of acute systemic toxicity in mice; as these results have not yet been independently peer-reviewed, they are reported here as supporting context and will be cited formally once published. Second, although this study included monitoring for rebleeding at 30 min and at 24 h post-procedure, longer-term follow-up (72 h to 7 days) is warranted in future studies, particularly under sustained antiplatelet therapy. Third, the efficacy of FHS in heparin-induced coagulopathy models has not yet been evaluated; this work is currently ongoing. Fourth, Axiostat^®^ was selected as the reference material because it is a commercially available chitosan-based hemostatic product of similar composition to FHS, enabling direct within-class comparison; comparison with agents of different classes is planned for future studies.

## 5. Conclusions

This study comprehensively evaluated the hemostatic efficacy of FHS in three experimental models (rabbit ear incision, and femoral artery puncture in Wistar rats and New Zealand White rabbits) under both normal physiological conditions and after aspirin administration. FHS demonstrated significantly superior hemostatic performance compared with medical gauze and a performance not significantly different from that of Axiostat^®^ across all three models.

Hemostatic efficacy was consistent across both superficial and deep arterial injury models and was not substantially compromised by aspirin administration. The hemostatic mechanism is primarily based on rapid blood fluid absorption, concentration of coagulation components, electrostatic interactions with blood cells, and physical occlusion at the injury site, without complete dependence on platelet aggregation function.

FHS represents a promising topical hemostatic agent, particularly in situations involving difficult-to-control hemorrhage or in patients receiving antiplatelet therapy. Future studies should evaluate toxicological profiles, in vivo biodegradability, biocompatibility, and efficacy in more severe coagulopathy models to further support the clinical translation of chitosan-based hemostatic biomaterials.

## Figures and Tables

**Table 1:** Comparison of physicochemical characteristics of FHS and Axiostat^®^.

Parameter	FHS	Axiostat^®^
Degree of deacetylation (DDA)		
—H NMR spectroscopy	73.57 ± 5.96%	73.9 ± 6.0%
—Acid–base titration	74.44%	75.75%
—UV spectrophotometry	87.7%	89.3%
Tensile strength (MPa)	23.22	13.12
Fluid absorption capacity (g/g)	24.8	26.9
Pore size range (µm)	40–170	50–300

**Table 2:** Time to hemostasis and blood loss in the rabbit ear incision model (New Zealand White rabbits), with and without aspirin treatment.

Material	Without Aspirin Time (s)	Without Aspirin Blood Loss (mg)	With Aspirin Time (s)	With Aspirin Blood Loss (mg)	Fold Increase (Time; Blood Loss)
Medical gauze	162.1 ± 9.9	208.3 ± 10.3	293.7 ± 18.5	504.8 ± 29.7	1.81; 2.41
FHS a,b	68.8 ± 5.1	62.6 ± 6.4	131.3 ± 10.3	155.2 ± 12.0	1.91; 2.48
Axiostat^®^ a,b	69.1 ± 5.1	63.7 ± 5.4	132.5 ± 8.6	153.8 ± 11.6	1.92; 2.42
Reduction vs. gauze—FHS (%)	57.6	69.9	55.3	69.3	—
Reduction vs. gauze—Axiostat^®^ (%)	57.4	69.4	54.9	69.5	—

Data are presented as mean ± SD. a: *p* < 0.001 vs. medical gauze (same condition); b: *p* < 0.01 vs. medical gauze (aspirin-treated condition). Welch’s independent-samples *t*-test. No statistically significant difference between FHS and Axiostat^®^ in either condition (*p* > 0.05). All aspirin-treated values were significantly different from non-aspirin values within the same material (*p* < 0.01). “—” indicates not applicable; the fold-increase metric applies only to the absolute time-to-hemostasis and blood-loss values (rows above), not to the percentage-reduction rows.

**Table 3:** Time to hemostasis and blood loss in the femoral artery puncture model—Wistar rats.

Material	Without Aspirin Time (s)	Without Aspirin Blood Loss (mg)	With Aspirin Time (s)	With Aspirin Blood Loss (mg)	Fold Increase (Time; Blood Loss)
Medical gauze	487.8 ± 81.7	864.6 ± 67.5	756.6 ± 35.4	5118.6 ± 178.5	1.55; 5.92
FHS a,b	171.5 ± 18.8	207.9 ± 15.1	255.3 ± 18.6	1277.6 ± 101.3	1.49; 6.15
Axiostat^®^ a,b	175.1 ± 17.8	211.2 ± 14.5	259.9 ± 15.5	1289.4 ± 107.9	1.48; 6.11
Reduction vs. gauze—FHS (%)	64.8	76.0	66.3	75.0	—
Reduction vs. gauze—Axiostat^®^ (%)	64.1	75.6	65.6	74.8	—

Data are presented as mean ± SD. a: *p* < 0.001 vs. medical gauze (same condition); b: *p* < 0.01 vs. medical gauze (aspirin-treated condition). Welch’s independent-samples *t*-test. No statistically significant difference between FHS and Axiostat^®^ in either condition (*p* > 0.05). All aspirin-treated values were significantly different from non-aspirin values within the same material (*p* < 0.01). “—” indicates not applicable; the fold-increase metric applies only to the absolute time-to-hemostasis and blood-loss values (rows above), not to the percentage-reduction rows.

**Table 4:** Time to hemostasis and blood loss in the femoral artery puncture model—New Zealand White rabbits.

Material	Without Aspirin Time (s)	Without Aspirin Blood Loss (mg)	With Aspirin Time (s)	With Aspirin Blood Loss (mg)	Fold Increase (Time; Blood Loss)
Medical gauze	508.8 ± 89.3	1131.6 ± 98.3	879.5 ± 60.3	5720.5 ± 328.3	1.73; 5.06
FHS a,b	192.8 ± 20.6	278.8 ± 23.4	335.3 ± 16.4	1397.7 ± 46.1	1.74; 5.01
Axiostat^®^ a,b	187.2 ± 19.5	282.4 ± 19.7	329.7 ± 25.8	1419.5 ± 32.2	1.76; 5.03
Reduction vs. gauze—FHS (%)	62.1	75.4	61.8	75.6	—
Reduction vs. gauze—Axiostat^®^ (%)	63.2	75.0	62.5	75.2	—

Data are presented as mean ± SD. a: *p* < 0.001 vs. medical gauze (same condition); b: *p* < 0.01 vs. medical gauze (aspirin-treated condition). Welch’s independent-samples *t*-test. No statistically significant difference between FHS and Axiostat^®^ in either condition (*p* > 0.05). All aspirin-treated values were significantly different from non-aspirin values within the same material (*p* < 0.01). “—” indicates not applicable; the fold-increase metric applies only to the absolute time-to-hemostasis and blood-loss values (rows above), not to the percentage-reduction rows.

## Data Availability

The data presented in this study are available on request from the corresponding author.

## Supplementary Materials

The following supporting information can be downloaded at: https://www.mdpi.com/article/10.3390/jfb17070349/s1, Table S1: Institutional technical specification for FHS (11 quality-control parameters); Figure S1: Representative intraoperative photographs across the three bleeding models.

## Abbreviations

The following abbreviations are used in this manuscript:

FHS	Field Hemostatic Sponge
COX-1	Cyclooxygenase-1
TXA2	Thromboxane A2
DDA	Degree of deacetylation
SEM	Scanning electron microscopy
SD	Standard deviation
SPSS	Statistical Package for the Social Sciences
TLR2	Toll-like receptor 2
3Rs	Replacement, Reduction, Refinement
