# Supplementary material for: Preclinical Evaluation of a Chitosan-Based Field Hemostatic Sponge in Arterial Bleeding Models with and Without Aspirin Administration
Source: J Funct Biomater. 2026 Jul 18;17(7):349. doi: 10.3390/jfb17070349 (PMC13412471; doi:10.3390/jfb17070349)
Supplement: Supplementary file 1 [file jfb-17-00349-s001.zip › jfb-4392232-supplementary.pdf]

Table S1. Institutional technical specification for FHS (11 quality-control parameters), per Decision No. 730/QĐ-TTNĐV (2 March 2024), Vietnam–Russia Tropical Centre.

Quality parameter	Acceptance criterion
Appearance	White to pale-yellow, uniform sponge; free of wet patches
Dimensional tolerance vs. 8 × 8 cm (%, max)	5.0
Weight loss on drying (%, max)	35.0
Extract solution pH	5.0–7.0
Fluid absorption ratio, vs. dry sponge weight (min)	20.0
Bacterial endotoxin (EU/sheet, max)	20.0
Tensile strength of sponge (N, min)	5.0
Package seal strength, dry condition (N, min)	1.2
Package seal strength, wet condition (N, min)	1.5
Package seal integrity	No leakage observed
Sponge unit weight (g)	2.8–3.4

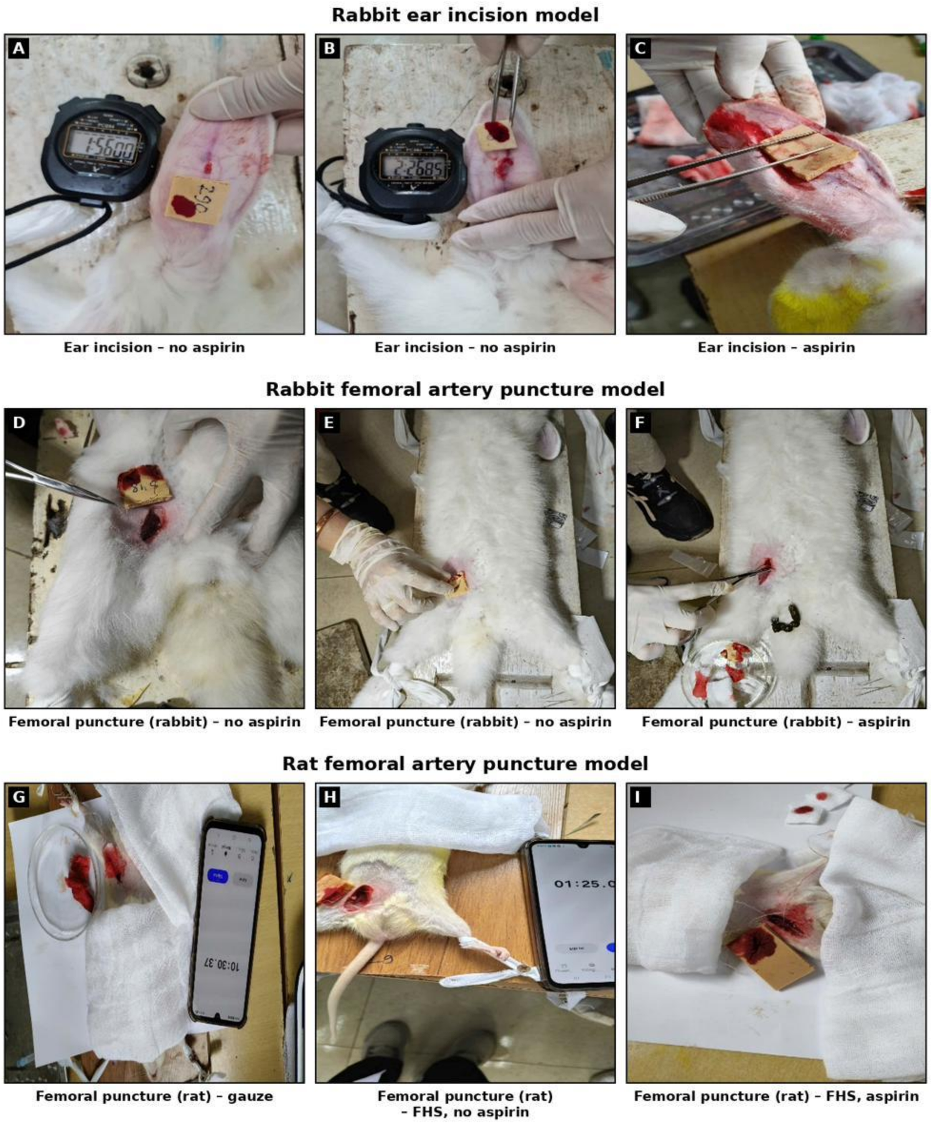

Figure S1. Representative intraoperative photographs across the bleeding models — rabbit ear incision (A–C), rabbit femoral artery puncture (D–F), and rat femoral artery puncture (G–I) — with and without aspirin pretreatment.
